# Professor Behring's "Tulase"—New Remedy for Consumption

**Published:** 1906-09-15

**Authors:** 


### Professor Behring's "Tulase."—New Remedy for Consumption.

Professor Von Behring's long looked for announcement on the cure of consumption by a preparation with which he has experimented was made last week. There are three methods of producing immunisation. "Jennerisation," a term adopted from the great Jenner who discovered vaccination, has been found efficacious against the Loeffler Bacillus, and as an antitubercular vaccine, when the vaccine obtained from a human source was applied to cows. "Mithridatism" is the name applied to another method of creating in the body a resistant action to poisons derived from organisms. Koch showed its power when he succeeded in habituating human beings to his tuberculine. This is an indispensable process in the manufacture of antitoxic serums. The anti-bodies are the product of the activity of the cells and living organs, and are capable of creating ACTIVE immunisation, but not of protecting against living bacilli. Persons in whom tolerance to tuberculine has been established are not immune against the tuberculosis caused by Koch's bacillus. Behring has succeeded in obtaining from the tubercle bacillus another toxin differing from Koch's tuberculin to which he applies the name "Tulase," which continues the process of immunisation where Koch's tuberculin left off. The third process of immunisation is by serum-therapy, and acts without any cellular agency, the cells remaining completely PASSIVE and taking no share whatever in bringing about the desirable result. Antibacterial bodies develop in the blood of infected persons who get well spontaneously, as in the case, say, of diphtheria or scarlet fever. These bodies may either be the curative agents or they may be the agents which produce future immunisation. This affords an explanation of how one attack of infectious disease prevents a person from ever afterwards being again attacked by the same disease. In the case of diphtheria Behring did not find these anti-bacillary forces either in the blood or blood-serum, but he discovered a disinfecting mechanism resembling what occurs when iodoform is applied to wounds. The disinfectant and antiseptic efficacy of iodoform do not rest on its antibacterial, but on its antitoxic effect, and pus and infected exudates are made inoffensive by contact with iodoform provided the iodoform becomes dissociated under the influence of bacterial products. The soluble diphtheria toxin discovered by Roux is rendered harmless by the serum of mithridised animals. Therefore the action of mithridisation and of serum-therapeutic immunisation is accomplished by antitoxic antibodies. The process of "ACTIVE immunisation" is longer and more dangerous than the PASSIVE. Consequently the serum-therapeutic method of obtaining immunity against infectious diseases is the one to be preferred whenever it can be obtained, as, for instance, in the case of the treatment of diphtheria by the anti-diphtheric serum.

Now the crucial question is that, having discovered an active immunisation "which is recognised as practicable against bovines," is it possible to produce an antiphthisical serum-therapy? Behring does not know to what practical results his research may yet lead, but at present he thinks that "active immunisation" (mithridatism) is the preferable method, combined, perhaps, with a serum treatment to which he makes reference.

"Tulase" is a liquid substance perfectly clear, yet containing the bodily substance (somatic) of Koch's bacillus (responding to the Ziehl and Gram methods of staining tubercle) prepared in a very complicated manner by treating tubercle bacilli with chloral. Its action is to modify the TC, and it acts when introduced either intravenously or subcutaneously or is administered through the stomach. The cells absorb the TC and transform it into the hypothetical substance TX. This substance produces immunity to tubercle bacillus and a hyper-sensitiveness to Koch's tuberculin. In sound persons "tulase" produces immunity in four months. The TC becomes TX more rapidly in persons already affected with tubercle, and hence the curative effect of "tulase." Its efficiency has been demonstrated on sheep with localised tubercular affections in the eye, on the skin, and in the lungs. The best results were obtained when the tulase was administered by the stomach in the form of an immunising milk. Behring is not keen at present to place this remedy in the hands of private practitioners, and until the exact doses and the best methods of treatment are ascertained it will not be sold. Hospital clinics will be supplied with the material only under certain conditions, a special course of instruction in Von Behring's laboratories at Marburg being one of the essential conditions.

